# Association between EGF +61 G/A and glioma risk in a Chinese population

**DOI:** 10.1186/1471-2407-10-221

**Published:** 2010-05-21

**Authors:** Shujie Wang, Yao Zhao, Zhenchao Ruan, Hongyan Chen, Weiwei Fan, Juxiang Chen, Qihan Wu, Ji Qian, Tianbao Zhang, Yan Huang, Daru Lu

**Affiliations:** 1 State Key Laboratory of Genetic Engineering, Institute of Genetics, School of Life Sciences, Fudan University, Shanghai 200433, China; 2 Neurosurgery Department of Huashan Hospital, Fudan University, Shanghai 200040, China; 3 State Key Laboratory of Genetic Engineering and Key Laboratory of Contemporary Anthropology, School of Life Sciences, Fudan University, Shanghai 200433, China; 4 Department of Neurosurgery, ChangZheng Hospital, Second Military Medical University, Shanghai Neurosurgical Institute, Shanghai 200003, China; 5 School of Life Science, East China Normal University, Shanghai 200062, China; 6 Department of Toxicology, Shanghai Second Military Medical University, Shanghai 200433, China

## Abstract

**Background:**

Epidermal growth factor (EGF) is critical in cancer process. EGF and EGF receptor (EGFR) interaction plays a pivotal role in cell proliferation, differentiation, and tumorigenesis of epithelial tissues. Variations of the EGF +61G/A (rs4444903) may lead to an alteration in EGF production and/or activity, which can result in individual susceptibility to brain glioma. The purpose of this study was to investigate the potential association between EGF +61G/A and brain glioma in a Chinese population.

**Methods:**

In this study, we analyzed single nucleotide polymorphism of EGF +61G/A in 677 patients with glioma and 698 gender- and age-matched controls. Genotyping was performed by polymerase chain reaction-ligation detection reaction (PCR-LDR) method.

**Results:**

The A allele (minor Allele) was 33.0% in cases and 27.3% in controls. The additive model was more powerful to reveal the association in our study than that of recessive and dominant model. Our data showed the genotype G/A and A/A was associated with increased risk for glioma (adjusted OR = 1.48, 95%CI: 1.17-1.87, p = 0.001 for G/A, adjusted OR = 1.81, 95%CI: 1.20-2.72, p = 0.005 for A/A, respectively), and for glioblastoma (adjusted OR = 1.51, 95%CI: 1.06-2.17, p = 0.024 and adjusted OR = 2.35, 95%CI: 1.34-4.15, p = 0.003, respectively). The A allele significantly increased glioma risk (OR = 1.31, 95%CI: 1.11-1.55, p = 0.001). The additive model (G/G vs G/A vs A/A) showed that both G/A and A/A genotype increased glioma risk (adjusted OR = 1.40, 95% CI: 1.17-1.66, p = 0.0002).G/A and A/A genotypes or EGF +61 A allele increased risk in both low and high WHO grade glioma. Non-smokers with G/A and A/A genotype showed increased glioma risk compared with G/G genotype (adjusted OR = 1.72, 95%CI: 1.29-2.30, p = 0.0002 and adjusted OR = 1.81, 95%CI: 1.10-2.99, p = 0.020, respectively). This association was not found in ever- or current-smokers.

**Conclusions:**

Our study indicated that G/A and A/A genotypes or EGF +61 A allele were associated with higher glioma risk in Chinese. This is in contrast with previous studies which reported G allele as a risk factor of glioma in Caucasian. The role of EGF +61 A/G polymorphism in glioma susceptibility needs further investigation.

## Background

Gliomas are a particularly lethal solid tumor arising from support cells in the central nervous system, which can be divided into astrocytic tumors, oligodendrogliomas, and oligoastrocytomas. These are then graded into 4 histological degrees of malignancy, according to the WHO classification [1]. Oligodendrogliomas and oligoastrocytomas are tiered into grade II, and anaplastic is grade III lesions. Astrocytomas, which are tumors composed predominantly of neoplastic astrocytes, amount to 80-85% of all gliomas. It is graded from low (grade I) to high (grade IV), according to hallmarks of the tumor histological aberrations [2]. Grade IV astrocytomas are known as glioblastoma multiforme (GBM), the most common and lethal form of malignant gliomas. The median survival time of GBM was only 10 months for those no older than 65 and 3.5 months for 65 or older [3]. However, recent research indicated that age groups (<65 and ≥65 years) was not associated with survival time, with median survival 13 months and 15 months, respectively [4]. Despite the development of therapy technology, the death rate of glioblastoma patients decreases a little, which indicates that the survival time of the most common and aggressive form of glioma in adults is very poor [5]. Previous studies suggested that some factors like workplace, dietary, and other personal and residential exposures might result in gliomas [6], as well as the genetic factors such as single nucleotide polymorphisms (SNPs) [7].

Epidermal growth factor (EGF), first isolated in 1962 [8], is encoded by a single gene on chromosome 4q25-q27 [9]. EGF has a profound effect on the differentiation and is a potent mitogenic factor for a variety of cultured cells of both ectodermal and mesodermal origin [10]. Its receptor, EGFR, is one of the first human EGF receptor (HER) family members discovered. In normal brain development, EGFR-mediated signaling plays a critical role. The EGFR family and its ligands are variably expressed from embryogenesis, throughout brain development, and into adulthood. They are involved in the proliferation, migration, differentiation, and survival of all central nervous system cell types and their precursors [11].

EGFR ligands such as EGF are often overexpressed in gliomas [12]. Bello et al. [13] observed that the postoperative levels of EGF decreased in all patients who underwent surgery and the entity of the decrease seemed to be well correlated with the extent of the tumoral resection. Shahbazi et al. [14] tested genetic polymorphisms in EGF in 135 white European patients with malignant melanoma and in 99 healthy white European controls. They identified a 61A-G (rs4444903) substitution in the 5-prime untranslated region of the EGF gene. Cells from individuals homozygous for the 61A allele produced significantly less EGF than cells from 61G homozygote or A/G heterozygote. Compared with the A/A genotype, G/G was significantly associated with increased risk of malignant melanoma. However, association studies on the polymorphism in various cancers had shown conflicting results [15-19].

Recent study showed that this polymorphism may causes expression difference and enhances glioma susceptibility [20]. Vauleon et al. [21] showed that the 61 A/G polymorphism was functional, in that the G allele promoter was 40% more active than the A variant (p < 0.001). However, analysis of 209 GBM patients and 214 control subjects did not confirm that 61 A/G polymorphism was a significant risk factor for GBM, despite a trend for higher G/G frequency in the patients. Costa et al. [20] studied 197 glioma patients and 570 cancer-free individuals; they found that the G allele conferred higher risk for gliomas, glioblastomas, and oligodendrogliomas. G/G genotype was significantly associated with increased risk for gliomas. In order to clarify the significance of this genetic polymorphism in glioma susceptibility, we did a case-control study of 677 glioma patients and 698 cancer-free controls in a Chinese population.

## Methods

### Study population

The population in this case-control study was similar to Liu et al. [22]. 677 patients diagnosed with histopathologically confirmed glioma were enrolled. All subjects, including case and control groups, were genetically unrelated ethnic Han Chinese and were from Shanghai and the surrounding provinces (Zhejiang, Jiangsu and Anhui) in east China. Patients were consecutively recruited between October 2004 and May 2006 in the Department of Neurosurgery at Huashan Hospital of Fudan University (Shanghai, China) with no restrictions of age, gender and histology. The research protocol was reviewed and approved by the Fudan University Ethics Committee for Human Subject Research.

The 698 cancer-free control subjects included trauma outpatients (20%) from the Emergency Medical Centre and hospital visitors (80%) who came to the health examination clinic for an annual check-up at the same hospital (Huashan Hospital) during the same period. The exclusion criteria for healthy subjects included central nervous system-related disease, self-reported history of any cancer and previous radiotherapy and chemotherapy for unknown disease conditions. All the control subjects were frequency matched to the cases on age (± 5 years), gender and residence area (urban or rural).

Each eligible subject was interviewed by a trained personnel who was not aware of the case and control with a structured questionnaire to obtain detailed information on demographic factors, family history of cancer (fmc), smoking status, and health characteristics. Fmc was defined as any self-reported cancer in the first-degree relatives (parents, siblings, or children). Never-smokers were defined as those who had smoked less than one cigarette per day and less than 1 year in their lifetime. Smokers were classified into ever-smokers and current-smokers. As a result, 806 glioma cases and 910 control subjects were recruited without the restrictions of age, sex and glioma histology. Among all participants, DNA samples and questionnaires were available from 677 cases and 698 control subjects representing an 84.0% and 76.7% of all eligible case and control subjects, respectively. A blood specimen was collected from each subject after the informed consent was obtained.

### DNA extraction and genotyping

3-5 ml venous blood was collected from each subject. EDTA-containing tubes were used to collect blood samples and then Qiagen Blood Kit (Qiagen, Chatsworth, CA, USA) was applied to extract genomic DNA. Polymerase chain reaction-ligation detection reaction (PCR-LDR) method was used to perform the genotyping. EGF+61 specific primers were: forward primer 5'-TAAAGGAAAGGAGGTGGAGCC-3', and reverse primer 5'-TGTGACAGAGCAAGGCAAAGG-3'. PCR was conducted on the ABI 9600 (Applied Biosystems, Foster City,CA,USA) in a system with total volume of 15 μl containing 1 ul genomic DNA, 1.5 μl 10×PCR Buffer, 0.13 μM each primer, 0.2 mM dNTP, 0.25 μl Taq DNA polymerase (Qlagen Gmbh, Hllden, Germany) and 7.5 μl H_2_O. The cycling parameters were: 94°C for 1 min; 35 cycles at 94°C for 10 s, 56°C for 20 s, 72°C for 40 s; and a final extension step at 72°C for 3 min. The probes for LDR were: 5'-P-GCTGGAACTTTCCATCAGTTCT-FAM-3'with common phosphorylated 5'- end and 6-carboxyXuorescein(FAM) labeled 3'-end, the G-specific probe 5' TTCAGCCCCAATCCAAGGGATGTG-3', the A-specific probe 5'-tttttTTCAGCCCCAATCCAAGGGATGTA-3'. For each PCR product, the ligation reaction was performed in a final volume of 10 ul including 2 μl of PCR product, 1 μl 10×Taq DNA ligase buffer, 0.02 μM of probe mixture, 5 U Taq DNA ligase (New England Biolabs, Beverly, Mass, USA) and 6 μl H_2_O. The LDR parameters were as follows: 25 cycles at 94°C for 30 s and 55°C for 4 min. The LDR reaction products were analyzed on ABI 377 DNA Sequencer (Applied Biosystems). To confirm the accuracy of PCR-LDR genotyping method, direct DNA sequencing of randomly selected PCR products was performed. The proportion of the sequencing samples were about 5%. The results of the PCR-LDR genotyping showed 100% concordance to direct DNA sequencing of the randomly selected PCR products.

### Statistical analysis

The Fisher's exact Chi-square test was first used to compare the frequency distribution of age, gender, smoking status and fmc between cases and controls. Hardy-Weinberg equilibrium (HWE) was tested by the χ2-test for goodness of fit using a web-based program http://ihg.gsf.de/cgi-bin/hw/hwa1.pl. To evaluate the association between EGF+61 G/A polymorphism and glioma risk, odds ratios (ORs) and 95% confidence intervals (CIs) were estimated by multivariate logistic regression analyses, adjusted by age and sex, or adjusted by age, sex, fmc, or adjusted by age, sex, smoking status and fmc, respectively. The genotype-smoking interaction was calculated by introducing an interaction term into the multivariate logistic regression which was adjusted by age, sex, fmc, smoking status and genotype. The patients were stratified into three subgroups according to histology: glioblastoma, astrocytomas (including diffuse astrocytomas, anaplastic astrocytomas or other astrocytomas except for glioblastoma) and other gliomas (including oligodendroglimas, enpendymomas or mixed glioma). Patients were also grouped with WHO grade to glioma: WHO I, WHO II, WHO III and WHO IV. Subgroup analysis according to histology and WHO grade were performed to estimate the specific ORs. Analyses were performed using the software SPSS 16.0 (SPSS Inc., Chicago, IL, USA). All *P *values were two-sided, and a *P *value < 0.05 was considered significant. QUANTO (version 1.2.4) software was used to calculate the statistic power and sample size. The power was computed as the probability of detecting an association between the EGF +61A/G polymorphism and glioma at the 0.05 significance level for the two-sided test, assuming an odds ratio of 1.2, 1.3, 1,4 in different genetic models, respectively. The sample size was calculated with the two-sided test in additive model, under the condition of 1.03 controls per case, the power and minor allele frequency (MAF) equal to 0.8 and 0.273, respectively.

## Results

### Characteristics of the study population

677 cases and 698 cancer-free controls were enrolled in our study. The genotyping was successful for EGF +61A/G in 672 glioma patients and 693 controls. The distribution of EGF+61 allele frequencies in the control group was in Hardy-Weinberg equilibrium (p>0.999). The characteristics of case patients and control subjects are summarized in Table 1. The mean age was 41.6 years (± 16.3 years, ranging from 2-79 yeas) for the cases and 39.6 years (± 18.3 years, ranging from 1-86 years) for the controls. Among 677 case patients, 256 (37.8%) had astrocytomas, 220 (32.5%) had glioblastoma and 201 (29.7%) had other gliomas. There were no significant differences on age and gender between the cases and the controls (p = 0.355 for age stratification and 0.141 for gender). In addition, no significant differences between the cases and the controls in smoking status and fmc were found (Table 1). The A allele was 33.0% in cases and 27.3% in controls. The frequencies of the G/G, G/A, and A/A genotypes of EGF +61 G/A were 52.8%, 39.8% and 7.4% in controls, and 44.3%, 45.4% and 10.3% in cases, respectively. The genotyping result showed that in this Chinese population the frequency of EGF+61G was higher than that of Caucasians. This is in accordance with the HapMap result (Table 2), with G allele frequency is higher in Asian, but lower in Caucasian compared with A allele.

**Table 1:** Charcteristics of selected patients with glioma and controls

Variable	Patients (n = 677) No.(%)	Controls (n = 698) No.(%)	P value*
Gender			0.141
Male	400(59.1)	384(55.0)	
Female	277(40.9)	314(45.0)	
Age(years)			0.355
Children(≤18)	69(10.2)	60(8.6)	
Adults(>18)	608(89.8)	638(91.4)	
Smoking status^a^			0.093
Never	386(65.8)	454(65.3)	
Ever	72(12.3)	111(16.0)	
Current	129(22.0)	130(18.7)	
Family history of cancer			0.056
No	566(83.6)	609(87.2)	
Yes	111(16.4)	89(12.8)	
Histology			
Astrocytomas^b^	256(37.8)		
Glioblastoma	220(32.5)		
Other gliomas^c^	201(29.7)		
WHO			
WHOI	61(9.0)		
WHOII	236(34.9)		
WHOIII	118(17.4)		
WHOIV	262(38.7)		

*Two-sides χ^2 ^test

a Smoking status information was absent for 90 patients and 3 controls

b Astrocytomas including diffuse astrocytomas, anaplastic astrocytomas, and other astrocytomas except for glioblastoma.

c Other gliomas including oligodendrogliomas, ependymomas, medulloblastoma, gliomatosis cerebri or mixed gliomas

**Table 2:** Distribution of EGF +61 G/A polymorphism in healthy control populations according to NIEHS and previous research

	N	Genotypes			Alleles		ethnicity
			
		GG(%)	GA(%)	AA(%)	G(%)	A(%)	
**Present study**	693	366(52.8)	276(39.8)	51(7.4)	1008(72.7)	378(27.3)	Asian
**Previous study**							
**Chinese**[32]	194	92(47.4)	84(44.3)	16(8.4)	270(69.6)	118(30.4)	Asian
**Chinese**[47]	212	100(47.2)	93(43.9)	19(9.0)	293(69.1)	131(30.9)	Asian
**Chinese**[48]	660	314(47.6)	289(43.8)	57(8.6)	917(69.5)	403(30.5)	Asian
**Chinese**[49]	206	98(47.6)	89(43.2)	19(9.2)	285(69.2)	127(30.8)	Asian
**Chinese**[50]	186	96(51.6)	73(39.3)	17(9.1)	265(71.2)	107(28.8)	Asian
**Chinese**[51]	654	328(50.2)	264(40.4)	62(9.5)	920(70.3)	388(29.7)	Asian
**Japanese**[18]	450	215(47.8)	188(41.8)	47(10.4)	618(68.7)	282(31.3)	Asian
**Korean**[31]	169	87(51.5)	68(40.2)	14(8.3)	242(71.6)	96(28.4)	Asian
**Korean**[35]	432	198(45.8)	185(42.8)	49(11.3)	581(67.2)	283(32.8)	Asian
**Mean for Asian**^**#**^		48.5(46.9-50.1)	42.2(40.8-43.6)	9.3(8.6-10.1)	69.6(68.6-70.6)	30.4(29.4-31.4)	
**Indian**[15]*	190	34(17.9)	87(45.8)	69(36.3)	155(40.8)	225(59.2)	Indian
**Danish**[52]*	81	15(19.0)	39(48.0)	27(33.0)	69(43.0)	93(57.0)	Caucasian
**British**[53]*	669	112(16.7)	338(50.5)	219(32.8)	562(42.0)	776(58.0)	Caucasian
**American**[54]*	232	30(12.9)	118(50.9)	84(36.2)	178(38.4)	286(61.6)	Caucasian
**Portuguese**[20]*	570	131(23.0)	266(46.7)	173(30.3)	528(46.3)	612(53.7)	Caucasian
**Cadadian**[55]*	447	72(16.0)	201(45.0)	174(39.0)	345(38.6)	549(61.4)	Caucasian
**Italian**[56]*	255	45(17.6)	117(45.9)	93(36.5)	207(40)	303(60)	Caucasian
**Australian**[19]*	2646	446(16.9)	1317(49.8)	883(33.4)	2209(41.7)	3083(58.3)	Caucasian
**Portuguese**[57]*	389	89(23.0)	180(46.0)	120(31.0)	358(46.0)	420(54.0)	Caucasian
**Northern European**[14]*	99	20(20.2)	47(47.5)	32(32.3)	87(43.9)	111(56.1)	Caucasian
**# Mean for Caucasian**		18.3(16.1-20.6)	47.6(46.1-49.1)	34.1(32.1-36.1)	42.1(40.1-44.1)	57.9(55.9-59.9)	
**Brazilian**[58]		56(28.00)	92(46.00)	52(26.00)	204(51.00)	196(49.00)	Brazilian
**NIEHS data**							
**CHB GENO PANEL**	45	20(44.4)	17(37.8)	8(17.8)	57(63.3)	33(36.7)	Asian
**JPT GENO PANEL**	44	24(54.5)	19(43.2)	1(2.3)	67(76.1)	21(23.9)	Asian
**YRI GENO PANEL**	60	34(56.7)	24(40.0)	2(3.3)	92(76.7)	28(23.3)	Sub-Saharan African
CEU GENO PANEL*	60	10(16.7)	27(45.0)	23(38.3)	47(39.2)	73(60.8)	European

Genotype frequencies and corresponding allele frequencies for EGF t61 G/A were shown for analyzed populations (refSNP ID: rs4444903). NIEHS, National Institute of Environmental Health Sciences; CHB, Han Chinese in Beijing, China; JPT, Japanese in Tokyo, Japan; YRI, Yoruba in Ibadan, Nigeria; and CEU, Utah residents with northern and western European ancestry.

* P < 0.05, compared with the present study.

# Mean (95%CI)

### Association between EGF +61 SNP and risk of glioma

The genotype distributions of cases and control subjects are summarized in Table 3. Overall, the A allele was associated with increased risk for glioma compared with G allele (OR = 1.31, 95% CI: 1.11-1.55, p = 0.001).

**Table 3:** Analysis of association between EGF+61 polymorphism and risk of glioma

	Case(%) N = 672	Control(%) N = 693	Crude P-value	Crude OR(95%CI)	Adjusted P*-value	Adjusted OR*(95%CI)	Adjusted P**-value	Adjusted OR**(95%CI)
**Genotype**								
**GG**	298(44.34)	366(52.81)		1(Reference)		1(Reference)		1(Reference)
**GA**	305(45.39)	276(39.83)	**0.007**	**1.36(1.09-1.70)**	**0.005**	**1.38(1.10-1.73)**	**0.001**	**1.48(1.17-1.87)**
**AA**	69(10.27)	51(7.36)	**0.011**	**1.66(1.12-2.46)**	**0.012**	**1.660(1.12-2.46)**	**0.005**	**1.81(1.20-2.72)**
**Allele#**								
**G**	901(67.04)	1008(72.73)		1(Reference)				
**A**	443(32.96)	378(27.27)	**0.001**	**1.31(1.11-1.55)**				
**Dominant**								
GG	298(44.34)	366(52.81)		1(Reference)		1(Reference)		1(Reference)
GA+AA	374(55.66)	327(47.19)	**0.002**	**1.41(1.14-1.74)**	**0.001**	**1.42(1.15-1.76)**	**0.0002**	**1.53(1.22-1.91)**
**Recessive**								
GG+GA	603(89.73)	642(92.64)		1(Reference)		1(Reference)		1(Reference)
AA	69(10.27)	51(7.36)	0.059	1.44(0.99-2.10)	0.066	1.43(0.98-2.09)	**0.043**	**1.50(1.01-2.22)**
**Additive**								
GG	298(44.34)	366(52.81)	**0.001**	**1.32(1.12-1.56)**	**0.001**	**1.33(1.12-1.57)**	**0.0002**	**1.40(1.17-1.66)**
GA	305(45.39)	276(39.83)						
AA	69(10.27)	51(7.36)						

*adjusted for age and sex

**adjusted for age, sex, family history of cancer (fmc) and smoking status

# the adjusted OR was not appropriated for the allele comparison

The smoking status information of 93 subjects was missing. The sample size decreased when adjusting for sex, age, fmc and smoking status, and the number of covariates may affect the results. Too many covariates may result in overfitting the logistic regression model, as well as decrease precision of the estimation of the variable interested [23]. Thus, we calculated three ORs for every independent analysis with different adjustment strategies, which provided more information for different stratified population. Using G/G genotype as reference, A/A and G/A genotypes were both associated with increased risk for glioma (adjusted OR** = 1.81, 95%CI: 1.20-2.72 and adjusted OR** = 1.48, 95%CI: 1.17-1.87, respectively). A/A was associated with glioma risk adjusted for gender, age, smoking status, fmc (adjusted OR** = 1.50, 95%CI: 1.01-2.22, p = 0.043) under recessive genetic model, whereas it failed to reach significance in the univariate analysis (Table 3). Dominant genetic model showed that G/A+A/A was significantly associated with the increased risk of glioma in univariate analysis (OR = 1.41, 95%CI: 1.14-1.74, p = 0.002) and multivariate model (adjusted OR** = 1.53, 95%CI: 1.22-1.91, p = 0.0002). Additive model showed that from G/G to A/A the risk significantly increased (adjusted OR** = 1.40, 95%CI: 1.17-1.66, p = 0.0002).

### The power of additive model

Table 4 showed the statistic power to demonstrate the association between EGF +61 G/A polymorphism and glioma if the OR was 1.3, 1.4, 1.5 under dominant, recessive, log additive genetic model, respectively. Generally, the power increased with OR, and the log additive model was the most powerful under a fixed OR. Under the condition that MAF was 0.273, sample size was 672 (1.03 control per case), the significant value was 0.05 (type I error = 0.05), and the OR was 1.40 in additive model, the predicted power was 0.981. However, the power was 0.973 if OR = 1.53 in dominant model. Thus, the additive model is the most powerful genetic model to reveal the association in our study; we then used this model in the following stratification analysis. A statistical power greater than 80% was obtained if the sample size was over 323 and 537 in additive model with OR equal to 1.40 and 1.30, respectively.

**Table 4:** Power of different genetic models

Model	OR	1.3	1.4	1.5
Log additive		0.880	0.981	0.998
Dominant		0.677	0.873	0.962
Recessive		0.270	0.417	0.571

Power were calculated under the condition of Minor Allele Frequency (MAF) = 0.273, sample size = 672 (1.03 control per case), type I error(α) = 0.05.

### Association between EGF +61 SNP and risk of glioma stratified by smoking status

Smokers were grouped as never-, ever-, and current-smokers as described above. In never-smokers, the A allele showed an increased association with the risk of glioma compared with G allele (OR = 1.47, 95%CI: 1.19-1.81, p = 0.0003), but no association were found in ever- and current-smokers (Table 5). For the comparison of different genotypes, the significant association was found only in the never-smokers. Individuals with G/A and A/A genotype showed increased glioma risk compared with G/G genotype when adjusted for sex-, age-, fmc- , with OR** = 1.72, 95% CI:1.29-2.30, p = 0.0002 and OR** = 1.81, 95%CI: 1.10-2.99, p = 0.020, respectively (Table 5). The *P *trend for G/G vs G/A vs A/A was 0.0003, the glioma risk significantly increased under additive genetic model (adjusted OR** = 1.49, 95%CI: 1.20-1.84). No association of EGF +61 G/A and the glioma risk in ever-smokers and current-smokers were found for different genetic comparisons (Table 5). However, this might result from the small sample size for ever- and current-smokers. The p-value for EGF +61 G/A genotype-smoking interaction was 0.617. The result indicated that no significant interaction existed between EGF +61 G/A genotypes and smoking.

**Table 5:** Stratified analysis of association by Smoking status between the genotypes (alleles) and risk of glioma

			Case/control	Crude p-value	Crude OR(95%CI)	Adjusted P*-value	Adjusted OR*(95%CI)	Adjusted P**-value	Adjusted OR**(95%CI)
**Never**	genotype	GG	148/239		1(Reference)		1(Reference)		1(Reference)
		GA	193/180	**0.0002**	**1.73(1.30-2.31)**	**0.0002**	**1.73(1.29-2.31)**	**0.0002**	**1.72(1.29-2.30)**
		AA	40/35	**0.016**	**1.85(1.12-3.04)**	**0.018**	**1.82(1.11-3.00)**	**0.020**	**1.81(1.10-2.99)**
	Genetic model	additive		**0.0002**	**1.50(1.21-1.86)**	**0.0003**	**1.49(1.20-1.85)**	**0.0003**	**1.49(1.20-1.84)**
	Allele	G	489/658		1(Reference)	#			
		A	273/250	**0.0003**	**1.47(1.19-1.81)**				
**Ever**	genotype	GG	31/55		1(Reference)		1(Reference)		1(Reference)
		GA	35/45	0.311	1.38(0.74-2.57)	0.395	1.32(0.70-2.51)	0.527	1.23(0.64-2.36)
		AA	6/8	0.625	1.33(0.42-4.19)	0.905	1.07(0.34-3.43)	0.941	1.05(0.32-3.37)
	Genetic model	additive		0.359	1.25(0.78-2.00)	0.572	1.15(0.78-1.87)	0.684	1.11(0.68-1.81)
	Allele	G	97/155		1(Reference)	#			
		A	47/61	0.373	1.23(0.78-1.95)				
**Current**	genotype	GG	68/70		1(Reference)		1(Reference)		1(Reference)
		GA	45/51	0.718	0.91(0.54-1.53)	0.874	0.96(0.56-1.63)	0.878	0.96(0.56-1.63)
		AA	16/7	0.077	2.35(0.91-6.08)	0.092	2.28(0.88-5.96)	0.090	2.29(0.88-5.99)
	Genetic model	additive		0.275	1.23(0.85-1.80)	0.254	1.25(0.85-1.83)	0.249	1.25(0.85-1.84)
	Allele	G	181/191		1(Reference)	#			
		A	77/65	0.259	1.25(0.85-1.84)				

*adjusted for age and sex

**adjusted for age, sex and fmc

# the adjusted OR was not appropriated for the allele comparison

p-value for genotype-smoking interaction was 0.617

### Association between EGF +61 G/A and risk of glioma stratified by histology

G/A and A/A genotypes were significant risk factors in glioblastoma, with adjusted OR** = 1.51, 95%CI: 1.06-2.17, p = 0.024 and adjusted OR** = 2.35, 95%CI: 1.34-4.15, p = 0.003, respectively. No association was found in astrocytomas (Table 6). For other gliomas, which including oligodendroglimas, enpendymomas and mixed glioma, G/A was a risk factor in both univariant logistic regression (OR = 1.50, 95% CI:1.08-2.10, p = 0.017) and multivariant analysis (adjusted OR** = 1.96, 95% CI:1.08-3.58, p = 0.028). However, the A/A genotype significantly increased other gliomas risk in multivariant model (adjusted OR** = 1.71, 95% CI: 1.20-2.45, p = 0.003), but failed to reach significance in univariant analysis. In the additive genetic model analysis, the *P *trend for G/G vs G/A vs A/A showed that the risk significantly increased both in glioblastoma and other gliomas, with adjusted OR** = 1.53, 95% CI: 1.18-1.97, *P *trend = 0.001 and adjusted OR** = 1.51, 95% CI:1.17-1.95, *P *trend = 0.002, respectively. The allelic analysis showed that the A allele was a risk factor for glioblastoma (OR = 1.42, 95%CI: 1.13-1.79, p = 0.003) and other gliomas (OR = 1.37, 95%CI: 1.08-1.75, p = 0.009), but not a risk factor for astrocytomas (Table 6).

**Table 6:** Stratified analysis of association by histology between the genotypes (alleles) and risk of glioma

		Case/control	Crude p-value	Crude OR(95%CI)	Adjusted P*-value	Adjusted OR*(95%CI)	Adjusted P**-value	Adjusted OR**(95%CI)
**Astrocytoma**^**a**^	GG	121/366		1(Reference)		1(Reference)		1(Reference)
	GA	112/276	0.182	1.23(0.91-1.66)	0.186	1.23(0.91-1.66)	0.092	1.32(0.96-1.81)
	AA	22/51	0.335	1.31(0.76-2.24)	0.327	1.31(0.76-2.25)	0.285	1.37(0.77-2.43)
	**Additive**		0.153	1.18(0.94-1.47)	0.152	1.18(0.94-1.48)	0.089	1.23(0.97-1.56)
	G	354/1008		1(Reference)	#			
	A	156/378	0.155	1.18(0.94-1.47)				
**Glioblastoma**	GG	94/366		1(Reference)		1(Reference)		1(Reference)
	GA	99/276	**0.042**	**1.40(1.01-1.93)**	**0.020**	**1.49(1.06-2.08)**	**0.024**	**1.51(1.06-2.17)**
	AA	27/51	**0.006**	**2.06(1.23-3.46)**	**0.010**	**2.035(1.19-3.49)**	**0.003**	**2.354(1.34-4.15)**
	**Additive**		**0.003**	**1.42(1.13-1.79)**	**0.002**	**1.45(1.14-1.84)**	**0.001**	**1.53(1.18-1.97)**
	G	287/1008		1(Reference)	#			
	A	153/378	**0.003**	**1.42(1.13-1.79)**				
**Other gliomas**^**b**^	GG	83/366		1(Reference)		1(Reference)		1(Reference)
	GA	94/276	**0.017**	**1.50(1.08-2.10)**	**0.017**	**1.50(1.08-2.10)**	**0.028**	**1.96(1.08-3.58)**
	AA	20/51	0.059	1.73(0.98-3.06)	0.055	1.75(0.99-3.10)	**0.003**	**1.71(1.20-2.45)**
	**Additive**		**0.009**	**1.38(1.09-1.76)**	**0.008**	**1.39(1.09-1.77)**	**0.002**	**1.51(1.17-1.95)**
	G	260/1008		1(Reference)	#			
	A	134/378	**0.009**	**1.37(1.08-1.75)**				

*adjusted for age and sex

**adjusted for age, sex, fmc and smoking status

a Astrocytomas including diffuse astrocytomas, anaplastic astrocytomas, and other astrocytomas except for glioblastoma.

b Other gliomas including oligodendrogliomas, ependymomas, medulloblastoma, gliomatosis cerebri or mixed gliomas

# the adjusted OR was not appropriated for the allele comparison

### Association between EGF +61 G/A and risk of glioma stratified by WHO grade

G/A genotype was found to be associated with higher risk for grade II (adjusted OR** = 1.52, 95%CI: 1.09-2.13, p = 0.013) and grade IV glioma (adjusted OR** = 1.59, 95%CI: 1.14-2.22, p = 0.006). G/A genotype was also found to be associated with grade I glioma in multivariant model (adjusted OR** = 1.92, 95%CI: 1.03-3.61, p = 0.041). Importantly, A/A genotype increased the risk for grade IV by more than 2-fold (adjusted OR** = 2.65, 95%CI: 1.58-4.45, p = 0.0002).

In additive model, the glioma risk increased significantly in WHO grade I (adjusted OR** = 1.69, 95%CI: 1.09-2.61, *P *trend = 0.018), grade II (adjusted OR** = 1.37, 95%CI: 1.07-1.75, *P *trend = 0.012), and grade IV (adjusted OR** = 1.62, 95%CI: 1.28-2.04, *P *trend < 0.001). A allele was associated with glioma risk for grade II and IV (OR = 1.30, 95%CI: 1.03-1.62, p = 0.025 and OR = 1.50, 95%CI: 1.22-1.86, p = 0.0002, respectively).

Both G/A and A/A genotypes increased glioma risk for low grade (WHO I+ II) using G/G as reference (adjusted OR** = 1.59, 95%CI: 1.17-2.16, p = 0.003 and adjusted OR** = 1.78, 95%CI: 1.04-3.03, p = 0.034, respectively). In additive model, G/A and A/A genotype increased low grade glioma risk gradually (adjusted OR** = 1.43, 95%CI: 1.14-1.79, *P *trend = 0.002). The allelic analysis indicated that allele A was a risk factor for low grade glioma (OR = 1.32, 95%CI: 1.07-1.63, p = 0.008). G/A and A/A genotypes also increased high grade gliomas (WHO III+ IV) risk, as well as the result under additive genetic model (Table 7). A allele increased glioma risk in high grade glioma (OR = 1.30, 95%CI: 1.07-1.58, p = 0.007).

**Table 7:** Stratified analysis of association by WHO between the genotypes (alleles) and risk of glioma

		Case/control	Crude p-value	Crude OR(95%CI)	Adjusted P*-value	Adjusted OR*(95%CI)	Adjusted P**-value	Adjusted OR**(95%CI)
**I**	GG	25/366		1(Reference)		1(Reference)		1(Reference)
	GA	28/276	0.167	1.49(0.85-2.60)	0.160	1.50(0.85-2.64)	0.041	1.92(1.03-3.61)
	AA	7/51	0.123	2.01(0.83-4.88)	0.102	2.11(0.86-5.16)	0.062	2.55(0.95-6.84)
**additive**			0.071	1.44(0.97-2.14)	0.060	1.47(0.98-2.19)	**0.018**	**1.69(1.09-2.61)**
	G	78/1008		1(Reference)	#			
	A	42/378	0.071	1.44(0.97-2.13)				
**II**	GG	103/366		1(Reference)		1(Reference)		1(Reference)
	GA	109/276	**0.033**	**1.40(1.03-1.92)**	**0.036**	**1.40(1.02-1.91)**	**0.013**	**1.52(1.09-2.13)**
	AA	22/51	0.125	1.53(0.89-2.65)	0.128	1.53(0.89-2.64)	0.098	1.63(0.91-2.92)
**additive**			**0.024**	**1.30(1.04-1.64)**	**0.026**	**1.30(1.03-1.63)**	**0.012**	**1.37(1.07-1.75)**
	G	315/1008		1(Reference)	#			
	A	153/378	**0.025**	**1.30(1.03-1.62)**				
**III**	GG	62/366	0.484	1(Reference)		1(Reference)		1(Reference)
	GA	49/276	0.821	1.05(0.70-1.57)	0.806	1.05(0.70-1.58)	0.583	1.13(0.74-1.73)
	AA	5/51	0.263	0.58(0.22-1.51)	0.264	0.58(0.22-1.51)	0.276	0.56(0.19-1.60)
**additive**			0.555	0.91(0.66-1.25)	0.564	0.91(0.66-1.256)	0.726	0.94(0.67-1.32)
	G	173/1008		1(Reference)	#			
	A	59/378	0.559	0.91(0.66-1.25)				
**IV**	GG	108/366		1(Reference)		1(Reference)		1(Reference)
	GA	119/276	**0.014**	**1.46(1.08-1.98)**	**0.006**	**1.55(1.13-2.12)**	**0.006**	**1.59(1.14-2.22)**
	AA	35/51	**0.001**	**2.33(1.44-3.76)**	**0.001**	**2.32(1.42-3.81)**	**0.0002**	**2.65(1.58-4.45)**
**additive**			**0.0002**	**1.50(1.21-1.87)**	**0.0002**	**1.53(1.23-1.91)**	**<0.001**	**1.62(1.28-2.04)**
	G	335/1008		1(Reference)	#			
	A	189/378	**0.0002**	**1.50(1.22-1.86)**				

**I+ II**	GG	128/366		1(Reference)		1(Reference)		1(Reference)
	GA	137/276	**0.017**	**1.42(1.07-1.89)**	**0.019**	**1.41(1.06-1.89)**	**0.003**	**1.59(1.17-2.16)**
	AA	29/51	0.056	**1.63(0.99-2.68)**	0.057	**1.63(0.99-2.68)**	**0.034**	**1.78(1.04-3.03)**
**additive**			**0.008**	**1.33(1.08-1.64)**	**0.009**	**1.33(1.07-1.64)**	**0.002**	**1.43(1.14-1.79)**
	G	393/1008		1(Reference)	#			
	A	195/378	**0.008**	**1.32(1.07-1.63)**				
**III+ IV**	GG	170/366		1(Reference)		1(Reference)		1(Reference)
	GA	168/276	**0.045**	**1.31(1.01-1.71)**	**0.025**	**1.36(1.04-1.78)**	**0.015**	**1.42(1.07-1.88)**
	AA	40/51	**0.023**	**1.69(1.07-2.65)**	**0.033**	**1.65(1.04-2.61)**	**0.016**	**1.79(1.11-2.89)**
**additive**			**0.007**	**1.30(1.07-1.58)**	**0.007**	**1.31(1.08-1.60)**	**0.003**	**1.37(1.11-1.68)**
	G	508/1008		1(Reference)	#			
	A	248/378	**0.007**	**1.30(1.07-1.58)**				

*adjusted for age and sex

**adjusted for age, sex, fmc and smoking status

# the adjusted OR was not appropriated for the allele comparison.

## Discussion

Carcinogenesis of glioma is considered to be a process affected by complicated factors. Environmental factors had been a focus in previous studies, however, except therapeutic ionizing radiation; no other environmental factors had been shown to be associated with glioma susceptibility [24]. Compared with other cancer types, knowledge about the role of genetic SNP in glioma is relatively limited.

EGF, a 6,045-Da single chain polypeptide which activates several signaling pathways such as ras/raf/MAPK and phosphatidylinositol-3-kinase (PI3K) [25], is of special importance in regulating cell proliferation, migration, adhesion, and inflammatory processes [26]. High expression of EGF had been detected in some cancer tissues, such as gallbladder cancer [15] and glioblastoma [27]. As an important receptor of EGF with high affinity, EGFR was often overexpressed in glioma cells. Amplification of EGFR gene was shown in half of the glioblastomas, as well as overexpression of EGFR mRNA and protein [28]. The overexpression related to a poorer prognosis in glioma patients [29]. These previous studies suggested that overexpression of EGF and EGFR might affect cell activities in brain tissues, abnormity of which may contribute to the glioma.

How does EGF +61 polymorphism affect the characteristic of EGF? This polymorphism had been proved to be functionally significant in individual variability of EGF expression. Shahbazi et al. [14] identified that cells from 61 A/A homozygous individuals produced significantly less EGF than cells from G/G (p = 0.0004) or G/A (p = 0.001) individuals. Tanabe et al. [30] showed that with EGF +61G, transcription product had significant longer (more than 2-fold) half-life than 61A allele. Serum EGF levels were 1.8-fold higher in G/G hepatocellular carcinoma patients with cirrhosis than A/A patients, and liver EGF levels were 2.4-fold higher in G/G patients than A/A patients. EGF+61G enhanced risk of hepatocellular carcinoma.

However, frequencies of EGF +61G allele were diverse among different subpopulation. Comparing to MAF of G allele in European population, the Chinese population occupy 0.633 of G allele (data shown in NCBI SNP database http://www.ncbi.nlm.nih.gov/SNP/snp_ref.cgi?rs = 4444903) (Table 2). We also compared the frequencies of the genotypes and alleles between control group in the present study and previous study on this SNP. Table 2 summarized the distribution of EGF +61 G/A polymorphism in different healthy control populations which fit in HWE except the study of Lim et al. [17]. The frequency of the EGF +61A allele in the present study was 27.3%, which was similar to the frequency observed in Chinese controls (30%-31%). The frequency of EGF +61A allele was about 23.9%-32.8% in Asians, which was different from that of Caucasians (53.7%-61.6%) (Table 2). Therefore, constitutive EGF expression may be different in different ethnic groups, which might lead to discrepancy results of association studies in different ethnic groups. Lim et al. [17] reported somewhat different distribution of EGF genotype in Korea healthy controls, the frequency of +61A allele was higher than that of other Asian populations, with a MAF = 0.4735. Although the discrepancy might be explained by their relatively small control sample size (n = 132), further studies were still needed to address the difference [31].

Previous study evaluated the influence of EGF+61 G/A to glioma risk, but no consensus had been reached [20,32]. The effects of EGF +61 G/A allele in various cancers were conflicting too. Shahbazi et al. [14] showed that G/G was significantly associated with increased malignant melanoma by examining 99 controls and 135 cases. Vishnoi et al. [33] found that G/G genotype of EGF +61A/G was significantly associated with increased gallbladder cancer risk by examining 126 gallbladder patients and 190 healthy controls; Goto et al. [34] found no association between EGF +61 G/A and gastric cancer but that EGF +61 A/A genotype showed a trend of protection by research on 202 patients and 454 healthy controls. Kang et al. [35] examined 432 lung cancer patients and 432 healthy age-and gender-matched control subjects and found that +61 A/A and +61 G/A genotypes were not significantly associated with the risk of lung cancer compared with the +61 G/G genotype. Tanabe et al. [30] examined 50 patients with cirrhosis and hepatocellular carcinoma and 148 patients with only cirrhosis. Logistic regression analysis demonstrated that the number of copies of G was significantly associated with increased hepatocellular carcinoma. However, the study of Gao et al. [36] didn't find association between the EGF +61 polymorphism and nasopharyngeal carcinoma in Chinese population by studying 173 patients and 206 controls, but A/A showed a trend to increase risk. By examining 383 breast cancer patients and 500 controls, Araujo et al. [37] found that carriers of G homozygous genotype had a lower risk for developing breast cancer (OR = 0.68, 95CI: 0.46-1.01). A lower risk for breast cancer in G/G carriers might be explained through EGF receptor internalization promoted by EGF.

According to Rosenthal's criteria [38], the link between EGF +61 G/A polymorphism and cancer is somehow plausible. It is essential to show that the change in the gene under study causes a relevant alteration in the function or level of the gene product, and EGF +61 G/A had been shown to be functional [14,30,39]. The EGF-EGFR signaling pathway performed an important role in regulating cell proliferation, migration, adhesion, and inflammatory processes [26], the correlation between this particular polymorphism and cancer have practical value. However, inconsistency existed in different studies, which may arise from many aspects. Cancer is a complex disease, EGF +61 G/A might play different roles in different kinds of cancers; Ethnic difference may also contribute to different disease susceptibility; Sample size must be large enough to be convincing. Larger studies will have greater power to detect an effect. Increasing the sample size results in an increased frequency of detecting marker/phenotype associations [40]. The number of patients in most of former studies about the association of EGF +61 G/A to cancers were less than 300, which might lead to a different result from our study involving 677 glioma patients and 698 healthy controls. In our paper reviewing process, a study in Northern-Chinese population failed to detect association of EGF+61 A/G and glioma [32], which might due to the small sample size (168 patients). However, stratification analysis in our study might have the decreased sample size to certain phenotypes. When stratified by WHO, the significant association of genotype A/A was only found in grade II and IV. The insignificant result in grade I and III might arise from the small sample size. Interestingly, stratification for different smoking status suggested A allele only increased the glioma risk in never-smokers. This might be explained of small sample size in ever- and current-smokers.

Our analysis showed the EGF +61 A allele may increase the risk of glioma, the stratification analysis indicated that A allele had no association with astrocytomas except for glioblastoma. Under different histology types of astrocytomas, glioblastoma, and other gliomas, the statistical power was 0.954, 0.952, and 0.920, respectively. For different grade of glioma, the power over 80% was only found in WHO IV, as well as in low grade and high grade gliomas. When adjusted for sex, age, fmc, smoking status, the glioma grade I risk significantly increased under additive genetic model(the power was only 0.743). However, Costa et al. [20] reported a significant association of the G variant with an increased risk of not only gliomas but also glioblastomas and oligodendroglial tumors in Portugal. Lanuti et al. [41] found that the association between EGF +61A/G and esophageal adenocarcinoma risk in earlier-stage patients might suggest that the G/G genotype was associated with a less aggressive phenotype, paralleling lung cancer where individuals with EGF pathway driven tumors seem to have fewer molecular alterations [42,43]; Consider the different allele frequency between Asians and Caucasians, further studies are needed to explore the role of EGF +61 G/A polymorphism in cancer development in different populations.

Cigarette smoking is a plausible behavioral exposure that might modulate glioma risk, but no overall association between glioma risk and cigarette smoking among either men or women [44,45]. The number of research on the role of smoking in glioma susceptibility was relatively small compared to other cancers such as lung cancer. Excision repair cross-complementing group 1 (ERCC1) is the lead enzyme in the nucleotide excision repair process. Stratified analyses revealed that the A/A genotype of *ERCC1 *8092C > A polymorphism was a risk factor in nonsmokers, but a protective factor in heavy smokers when compared with the C/C genotype [46]. No study had investigated the association between EGF polymorphism and the risk of glioma under different smoking status. In our study, the genotype-smoking interaction was insignificant. We also calculated the power of the stratification analysis by smoking status. The result showed that the power was 0.964 in never-smokers, which was of strong ability to test the association between EGF +61 G/A and glioma. However, in ever- and current-smokers, the power was 0.421 and 0.550, respectively. It is possible that the insignificant results in ever- and current-smokers might attributable to relatively small number of subjects. Additional studies with more subjects will be needed.

Our study included a relatively large sample size from a homogeneous population of the same ethnicity. However, several limitations in our study need to be addressed. The association between EGF +61 SNP (rs4444903) and risk of gliomas is biologically plausible, since EGF performs a very important role in the proliferation, migration, and differentiation [11]. However, only one SNP in EGF was examined in our study. It remains uncertain whether the rs4444903 is in strong LD with a causative variant located inside or near the EGF locus. Secondly, the association between EGF +61A/G and glioma risk was controversial in our study and Caucasions [20], Thus, studies with ethnically diverse populations are warranted to confirm our findings and to further elucidate the significance of the polymorphism in the development of gliomas. Finally, further functional experiments are therefore necessary to test the hypotheses.

## Conclusions

In conclusion, we demonstrated that the variant genotypes of G/A and A/A were associated with a significantly increased risk of glioma, especially glioblastma, when compared with wild-type homozygote G/G. Whether there were association between EGF +61 G/A and grade I and III glioma deserved further study, as well as EGF gene - smoking interaction. To our knowledge, this is the first study to investigate the potential association between the EGF +61 G/A polymorphism and gioma in a Chinese population from east China.

## Competing interests

The authors declare that they have no competing interests.

## Authors' contributions

SW carried out the data analysis, performed the further interpretation of data, drafted the manuscript, and mainly generated the tables. YZ collected the samples, co-worked in data analysis, as well as manuscript preparation. ZR participated in the calculation and table generation, and helped to draft the manuscript. HC, WF, JC, QW, JQ, TZ also contributed to sample collections, face-to-face interview of each participant, and manuscript editing. YH and DL organized all the research, provided advice for preparing the manuscript, final editing of the manuscript. All authors read and approved the final manuscript.

## Pre-publication history

The pre-publication history for this paper can be accessed here:

http://www.biomedcentral.com/1471-2407/10/221/prepub
